# Predicting and Co-Optimizing the Taste and Aroma of Green Tea During Spreading Using the TabPFN Model

**DOI:** 10.3390/foods15122069

**Published:** 2026-06-08

**Authors:** Haotian Qian, Xinyao Yang, Pengcheng Zheng, Shengpeng Wang, Rui Hu, Junyi Chen

**Affiliations:** 1 College of Intelligent Science and Engineering, Hubei Minzu University, Enshi 445000, China; 202530355@hbmzu.edu.cn (H.Q.); 202430323@hbmzu.edu.cn (X.Y.); 202310613@hbmzu.edu.cn (R.H.); 2 Institute of Fruit and Tea, Hubei Academy of Agricultural Sciences, Wuhan 430064, China; zpct@hbaas.ac.cn (P.Z.); wangshengpeng@hbaas.ac.cn (S.W.)

**Keywords:** green tea, spreading, non-targeted metabolomics, machine learning, TabPFN, process optimization

## Abstract

To investigate how spreading conditions affect green tea taste and aroma and to develop a generalizable prediction model from small data for process optimization, this study integrated SEM, non-targeted dual-omics, and TabPFN to systematically analyze Echa No. 10 spreading. A central composite design was used. Dehydration-induced mechanical stress altered cell membrane permeability, driving non-volatile taste compound transformation and volatile aroma release. Two chemical-sensory proxies, relative polyphenol-to-amino acid ratio (R-PAR) and floral intensity index (FII), were established using ultra-high performance liquid chromatography–high-resolution mass spectrometry (UHPLC-HRMS) and headspace solid-phase microextraction–gas chromatography–mass spectrometry (HS-SPME-GC-MS). A prediction model was built with these indicators and TabPFN. Multi-objective optimization yielded optimum conditions: initial moisture 76.8%, temperature 26.2 °C, relative humidity 61.5%, air speed 0.85 m/s, achieving R-PAR 0.465 and FII 125.70. Compared with response surface methodology (RSM), partial least squares regression (PLSR), and support vector regression (SVR), TabPFN showed prediction R^2^ of 0.81 and 0.77, showing favorable applicability and predictive capability on small-sample data. This study validates TabPFN’s suitability for small-sample tea processing modeling, quantifies the mapping between spreading and key taste/aroma metabolism, and provides a methodological foundation for digital precision and intelligent optimization in green tea production.

## 1. Introduction

Tea is the most widely consumed beverage raw material worldwide, characterized by rich product diversity and pronounced flavor differentiation. Depending on the processing method and degree of fermentation, tea can be classified into six major categories: green tea, black tea, oolong tea, white tea, yellow tea, and dark tea [1]. Among these, green tea stands out for its abundance of natural bioactive compounds such as tea polyphenols and amino acids, endowing it with significant health benefits and nutritional value [2,3]. According to the 2025 China Tea Production and Marketing Situation Analysis Report released by the China Tea Marketing Association [4], China’s total dry tea output reached 3,640,300 tons in 2025, of which green tea production amounted to 2,148,700 tons, accounting for 59.03% of the national total. Green tea thus represents the largest and most industrially established core tea category in China. Among the numerous evaluation criteria that determine the grade and market price of green tea, taste and aroma serve as the central quality factors, together contributing up to 55% of the total weighting in comprehensive sensory evaluation [5].

Spreading is the first key processing step for green tea and represents a critical window for shaping its taste and aroma quality [6]. As moisture dissipates, leaf cells undergo shrinkage and stomatal closure. This physical and mechanical stress increases cell membrane permeability, facilitating effective contact between endogenous hydrolases and their substrates [7]. These two processes effectively promote the hydrolysis of macromolecular substances such as proteins, polysaccharides, and glycosides, as well as the directed degradation of lipids, thereby eliminating grassy notes, generating characteristic aroma, and markedly enhancing umami and mellow taste [8].

In recent years, high-throughput metabolomics has provided powerful analytical tools for deciphering the evolution of taste and aroma compounds during tea processing [9]. Ultra-high performance liquid chromatography–high-resolution mass spectrometry (UHPLC-HRMS) has been widely adopted for the qualitative and quantitative analysis of non-volatile taste-related compounds such as catechins and free amino acids [10]. Meanwhile, headspace solid-phase microextraction coupled with gas chromatography–mass spectrometry (HS-SPME-GC-MS) serves as a core technique for the effective separation and identification of volatile aroma constituents [11]. Although some studies have employed metabolomics to characterize the taste and aroma profiles of tea [12,13], these efforts have largely focused on monitoring individual compound concentrations without establishing correlative models linking process parameters to taste and aroma attributes. This limitation hinders the precise control of green tea processing [14].

Machine learning techniques have been widely applied in the tea processing domain with considerable success. However, the accuracy of these models typically depends on large sample sizes, and their modeling capability becomes severely constrained in small-sample scenarios. Given that tea processing is restricted by seasonal harvesting windows and the high cost of wet chemistry experiments, only small-scale trials are usually feasible. The resulting small feature matrices can easily drive complex models into overfitting. The Tabular Prior-Data Fitted Network (TabPFN) is a machine learning model specifically designed for small-sample experimentation. Built upon the Transformer architecture, TabPFN undergoes offline meta-learning on massive synthetic tabular datasets, encoding complex statistical prior knowledge into its network parameters [15]. This architecture enables TabPFN to directly approximate Bayesian posterior inference through in-context learning, without the need for gradient updates or hyperparameter tuning for a given target task. It thereby circumvents the overfitting risk inherent in small-sample settings while ensuring that predictions at the extreme boundaries of process parameters remain consistent with biochemical reaction principles, offering a robust modeling solution for tea processing studies constrained by limited sample sizes.

Against the above background, this study takes the fresh leaves of Echa No. 10 as the research material. Scanning electron microscopy (SEM) was employed to observe microstructural changes under dehydration stress. Because floral notes constitute the core aroma profile of this specific cultivar, dual-omics techniques were applied to screen and define two core chemical-sensory proxies of its taste and aroma quality: the relative polyphenol-to-amino acid ratio (R-PAR) and the Floral Intensity Index (FII). On this basis, the TabPFN model, designed for small-sample scenarios, was introduced to construct a spreading quality prediction model with R-PAR and FII as proxy indicators and to perform multi-objective collaborative global process optimization. The performance of TabPFN was then benchmarked against mainstream modeling approaches to verify its advantages and predictive reliability under this specific process scenario, aiming to provide a novel modeling framework to support the digital precision processing of green tea. The novelty of this study lies in: (1) introducing the TabPFN model into the field of tea processing quality prediction for the first time, and (2) achieving multi-objective collaborative optimization of spreading process for both taste and aroma using dual-quality indicators jointly constructed by non-targeted metabolomics and volatilomics.

## 2. Materials and Methods

### 2.1. Experimental Materials and Equipment

#### 2.1.1. Tea Samples and Reagents

The fresh tea leaves used in this study were harvested from Xiaocun Township, Xianfeng County, Enshi Prefecture, Hubei Province, between 25 March and 15 April 2025. Picking was conducted daily at 8:00 a.m. and suspended on rainy days. The cultivar was Echa No. 10, and the picking standard was strictly controlled to one bud and two leaves. Chromatographic-grade methanol, acetonitrile, formic acid, and isopropanol, along with deuterated styrene (25 μg/mL), were purchased from Sigma-Aldrich (St Louis, MO, USA). The 2.5% glutaraldehyde fixative and graded dehydration reagents were obtained from Beijing Solarbio Science & Technology Co., Ltd. (Beijing, China). Liquid nitrogen (purity ≥ 99%) was supplied by a local gas supplier. Ultrapure water produced by a Milli-Q ultrapure water system (Millipore, Burlington, MA, USA) was used throughout all experiments.

#### 2.1.2. Experimental Equipment

**Figure 1:**
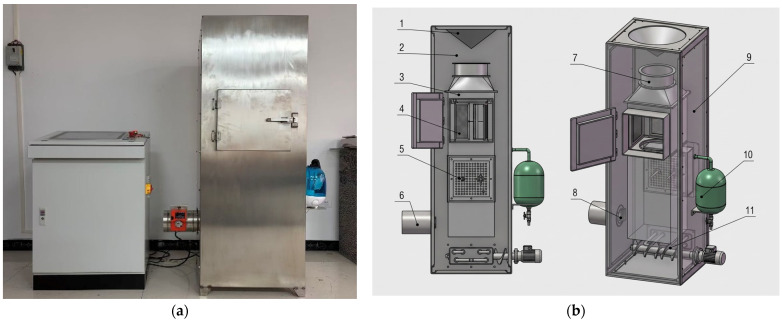
Multi-parameter controllable thin-layer spreading test bench: (**a**) Physical photograph; (**b**) Schematic diagram. 1. Air flow guiding device; 2. Test bench outer casing; 3. Test bench inner chamber; 4. Material compartment; 5. Sensor compartment; 6. Electric sealing valve; 7. Axial flow fan; 8. Dehumidification fan; 9. Test bench side panel; 10. Air humidifier; 11. Heating device.

The JDKY-I multi-parameter controllable thin-layer spreading test bench, designed and manufactured by Changchun Jida Scientific Instrument Equipment Co., Ltd. (Changchun, China), and manufactured in 2023, served as the simulation equipment for green tea spreading (see Figure 1). The equipment dimensions are as follows: Length 550 mm × Width 550 mm × Height 1800 mm. The technical parameters include: Air velocity ranging from 0 to 1 m/s with an accuracy of ±0.05 m/s; a temperature range from room temperature to 100 °C with an accuracy of ±1 °C; and a relative humidity range of 20% to 80% with an accuracy of ±2%.

Other equipment: An HC103 halogen moisture analyzer (Mettler Toledo, Greifensee, Switzerland), a Shimadzu ATX124R analytical balance with 0.1 mg readability (Shimadzu Corporation, Tokyo, Japan), a MAS-II Plus microwave synthesis/extraction workstation (Shanghai Xinyi Microwave Chemical Technology Co., Ltd., Shanghai, China), a Scilogex D3024R low-temperature high-speed centrifuge (Scilogex Scientific Instruments Co., Ltd., Rocky Hill, CT, USA), an Agilent 1100 high-performance liquid chromatograph equipped with a UV detector, an Agilent 7890 gas chromatograph coupled with an Agilent 5975 mass spectrometer together with the corresponding solid-phase microextraction fiber assembly (Agilent Technologies, Inc., Santa Clara, CA, USA), a Vanquish ultra-high performance liquid chromatograph and a Q Exactive™ HF high-resolution mass spectrometer (Thermo Fisher Scientific, Waltham, MA, USA), a JSM-6700F cold field emission scanning electron microscope (JEOL Ltd., Tokyo, Japan), and a Milli-Q ultrapure water system (Millipore).

### 2.2. Spreading Experiment

Utilizing Design-Expert software (version 8.0.6.1) in conjunction with the Central Composite Design (CCD) method, a four-factor, five-level spreading experiment comprising 21 runs was designed for green tea. The experimental factors included initial moisture content (matched to the nearest level, as the actual moisture of fresh leaves cannot be perfectly aligned with predefined values), spreading temperature, relative spreading humidity, and spreading airflow velocity. Each spreading treatment was repeated three times, yielding a total of 63 samples. The independent variables and their designated levels are detailed in Table 1.

The specific spreading procedures were conducted as follows: (1) A 5 g (±0.0001 g) sample of fresh tea leaves was extracted to determine the initial moisture content using a halogen moisture analyzer; this measurement was performed in triplicate. (2) A 0.6 kg batch of fresh tea leaves was placed into a multi-parameter controllable spreading apparatus. The experiment was executed according to the specific parameter levels outlined in Table 1. Once spreading commenced, samples were taken every hour to predict the moisture content using the gravimetric method. (3) When the predicted moisture content decreased to 71%, the sampling and weighing frequency was increased to every 30 min. The spreading process was deemed complete when the predicted moisture content reached 70%. Subsequently, the actual moisture content was determined in accordance with the national standard GB/T 8304-2002 (Tea—Determination of moisture content) [16]. (4) Following the methodology described in a previous study [17], the spread samples were subjected to microwave fixation at a power level of 6 kW. (5) The fixed samples were placed in a forced-air drying oven at 80 °C for 1 h, and then transferred to a desiccator to cool to room temperature for subsequent use. (6) The prepared samples were individually subjected to qualitative and quantitative analyses to determine the core taste and aroma compounds of the green tea. (7) Across all aforementioned steps, sampling was conducted using the quartering method, in reference to GB/T 8302-2002 (Tea—Sampling) [18]. Furthermore, the grinding of samples destined for quality component analysis was performed according to GB/T 8303-2002 (Tea—Preparation of ground sample and determination of dry matter content) [19]. For all subsequent physicochemical analyses, both UHPLC-HRMS and HS-SPME-GC-MS measurements for each spreading sample were performed in triplicate, and the arithmetic mean was used for subsequent data analysis and modeling.

### 2.3. Physicochemical Analysis

#### 2.3.1. Microstructural Observation via Scanning Electron Microscopy

To investigate the effects of spreading-induced stress on the histological structure of fresh tea leaves at the microphysical level, the microstructures of the treated samples were observed using SEM. The observations encompassed the overall structure of the epidermal cells (100×), the evolution of mechanical epidermal shrinkage (500×), and the distribution and initial state of the stomata (500×), as well as the high-magnification microscopic features of stress-induced stomatal closure (2000×). Furthermore, the analysis included the contractive structure of the vascular bundles in the tea stalks (50×), the curling and structural damage characteristics of the trichomes (2500×), and the textural alterations of the cuticular wax alongside the evolution of drought-induced microcracks (2500×). By comparatively analyzing the differences in these microstructural features under various spreading conditions, the extent to which the intensity of environmental stress degraded the physical structure of the tea cells was qualitatively demonstrated. The sample preparation and observation procedures were conducted according to standard scanning electron microscopy (SEM) preparation methods for biological samples [20].

#### 2.3.2. UHPLC-HRMS Methodology

The detection method was based on reference [21], with the following modifications tailored to the characteristics of fresh green tea leaves: Briefly, 100 mg of freeze-dried tea powder, previously ground in liquid nitrogen, was accurately weighed and mixed with 500 μL of an 80% aqueous methanol solution. The mixture was vortexed thoroughly and incubated in an ice bath for 5 min, followed by centrifugation at 15,000× *g* for 20 min at 4 °C. The resulting supernatant was diluted with ultrapure water to a final methanol concentration of 53% and centrifuged again. This final supernatant was collected for subsequent analysis. Simultaneously, quality control (QC) samples, prepared by mixing equal volumes of the experimental samples, and blank samples (53% methanol) were established.

Chromatographic and Mass Spectrometric Parameters: Chromatographic separation was performed using a Vanquish UHPLC system coupled with a Hypersil Gold C18 column (100 × 2.1 mm, 1.9 μm). The column temperature was maintained at 40 °C, the injection volume was set to 2 μL, and the flow rate was held constant at 0.2 mL/min. The mobile phase consisted of a 0.1% aqueous formic acid solution (A) and methanol (B). The elution gradient was programmed as follows: 0–1.5 min, maintained at 2% B; 1.5–3.0 min, linearly increased to 85% B; 3.0–10.0 min, further increased to 100% B. Mass spectrometry was conducted using a Q Exactive HF high-resolution mass spectrometer. Fragmentation data within an *m*/*z* range of 100–1500 were acquired in both positive and negative ion modes utilizing data-dependent acquisition (DDA).

Data Processing and Metabolite Identification: The raw instrumental data were extracted and aligned using XCMS software (Version 3.16.0), followed by qualitative identification against the NovoMetDB database. After filtering out unstable characteristic peaks with a coefficient of variation (CV) > 30% in the QC samples, precise qualitative identification and pathway annotation of the metabolites were achieved by integrating a high-quality local MS2 spectral library with public databases, including the Kyoto Encyclopedia of Genes and Genomes (KEGG), Human Metabolome Database (HMDB), and Lipid Metabolites and Pathways Strategy (LIPID MAPS).

#### 2.3.3. Calculation of R-PAR

The concentration ratio of polyphenols to free amino acids serves as a core theoretical index for evaluating the balance between astringency and umami, as well as the overall quality of tea infusions. Given the significant disparities in the ionization efficiencies of various compounds in UHPLC-HRMS data, directly comparing raw peak areas lacks statistical validity. Consequently, this study introduced the min-max normalization pretreatment strategy, a classic approach in metabolomic data analysis [22]. Based on this dimensionality reduction strategy, the objective function for R-PAR was constructed. Referring to previous studies on the taste profiles of green tea [23,24], from the untargeted mass spectrometry data of the experimental groups, the raw peak areas $x_{i,j}$ of the *m* core polyphenols and *n* core free amino acids, which decisively influence the astringent and umami qualities, were explicitly identified. Subsequently, the corresponding peak areas of the *m* + *n* compounds in the experimental samples were individually subjected to min-max normalization

(1) $$N_{i,j}=\frac{x_{i,j}-x_{i,min}}{x_{i,max}-x_{i,min}}$$

the comprehensive retention index of polyphenols $P_{j}$:

(2) $$P_{j}=\frac{1}{m}\sum_{i=1}^{m}N_{i,j}$$

the comprehensive retention index of amino acids $A_{j}$:

(3) $$A_{j}=\frac{1}{n}\sum_{i=1}^{n}N_{i,j}$$

the relative polyphenol-to-amino acid ratio (R-PAR, $R_{j}$):

(4) $$R_{j}=\frac{P_{j}}{A_{j}}$$

It should be noted that R-PAR is not a direct quantification of sensory taste, but rather a chemical-sensory proxy constructed on the basis of compounds with known taste contributions. It was determined that a lower R-PAR value indicates superior spreading quality, which aligns perfectly with the metabolic transformation objective of “suppressing bitterness and enhancing umami” during green tea processing [25]. Therefore, R-PAR can serve as a reliable chemical indicator for evaluating the evolution of taste quality under the present experimental conditions.

#### 2.3.4. HS-SPME-GC-MS Determination Method

The detection method was performed with slight modifications based on the previously reported literature to suit the characteristics of green tea [26]. Extraction and SPME Conditions: The tea samples were shredded, and 1.00 g of the solid sample was accurately weighed into a headspace vial. Subsequently, 20 μL of internal standard solution (deuterated styrene, CAS: 19361-62-7, 25 μg/mL in methanol) was added. The vial was equilibrated at 60 °C and 250 r/min for 15 min. The SPME fiber was then inserted into the headspace of the vial for extraction at 60 °C for 30 min. Finally, the fiber was inserted into the GC inlet and thermally desorbed at 260 °C for 5 min. GC-MS Parameters: Analysis was conducted using an Agilent 7890 GC coupled with an Agilent 5975 MS, equipped with an HP-5MS Ultra Inert capillary column (30 m × 0.25 mm × 0.25 μm, Agilent Technologies). The injection was performed in split mode (split ratio 25:1) with high-purity helium (99.999%) as the carrier gas at a constant flow rate of 1.0 mL/min. The oven temperature program was set as follows: initial temperature at 50 °C (held for 5 min), increased to 230 °C at a rate of 7 °C/min (held for 3 min), and finally increased to 320 °C at a rate of 40 °C/min (held for 5 min). The MS parameters were: ion source temperature 240 °C, quadrupole temperature 160 °C, ionization energy 70 eV, and mass scan range 50–500 *m*/*z*. Data Processing and Identification: Raw data were converted to .abf format via the Analysis Base File Converter. Peak picking, alignment, and integration were performed using MSDIAL software (version 4.60). Accurate identification of metabolites was achieved by matching mass spectra and Retention Index (RI) with the NIST 2020 database. Finally, the relative content ($C_{i}$) of each monomer was output based on the internal standard method to construct the volatile relative abundance matrix. This matrix served as the fundamental data input for the subsequent calculation of the Floral Intensity Index (FII), without further secondary conversion to absolute peak areas [27].

#### 2.3.5. Calculation of FII

Water-deficit stress during the green tea spreading process drastically induces carotenoid degradation and terpenoid metabolic pathways, prompting the fresh leaves to emit pronounced floral and fruity aromas. To eliminate environmental background noise in high-dimensional omics data and establish a direct mapping between physical abundance and human sensory perception, this study introduced the Relative Odor Activity Value (ROAV) quantitative method [28] to construct the FII, which exhibits high sensitivity to variations in the spreading process.

By referencing the standard odor thresholds of volatile aroma compounds in the aqueous phase from the literature [29], and integrating the relative content and olfactory threshold of each component, the ROAV for each aroma component was calculated, as detailed in the Equation.

(5) $$ROAV_{i}=\frac{C_{i}/T_{i}}{C_{max}/T_{max}}\times 100$$

where $C_{i}$ is the relative content (μg/kg) of the odor-active compound; $T_{i}$ is the standard olfactory threshold of that compound in the aqueous phase. $C_{max}$ and $T_{max}$ represent the relative content and the olfactory threshold, respectively, of the component exhibiting the maximum aroma contribution across the entire sample set.

To achieve global comparability of aroma quality among different spreading treatment groups, the ROAV of the component with the maximum overall aroma contribution across the entire sample set (i.e., the compound with the highest ratio of relative content to its corresponding olfactory threshold) was defined as 100. According to the consensus in flavor chemistry evaluation, combined with the globally normalized ROAV calculation logic of this study, when ROAV ≥ 1, the compound is determined to be a core odor-active compound that dictates the aroma profile of the sample; when 0.1<ROAV< 1, it is considered to exert a modifying effect on the overall aroma [30]. In this study, only the core key components satisfying the threshold of ROAV ≥ 1 were strictly selected and arithmetically summed to construct the FII. The formula is as follows:

(6) $$FII={ROAV}_{1}+R{OAV}_{2}+\cdots$$

It should be noted that the FII is a chemical-sensory proxy indicator calculated based on the weighting of relative odor activity values (ROAV). It aims to focus on the tea aroma characteristics contributed by key aroma-active compounds, thereby providing a highly sensitive quantitative objective function for subsequent high-throughput grid optimization based on the TabPFN machine learning model. In multi-objective collaborative optimization, a higher FII value indicates a superior characteristic floral aroma in the spread leaves. Although the FII cannot completely replace human sensory evaluation, it incorporates odor threshold information. Compared to evaluation methods that rely solely on compound concentrations, it can more scientifically and accurately reflect the dynamic changes in the perceived intensity of key floral aromas during the spreading process.

### 2.4. Modeling and Verification of Spreading Quality Based on Machine Learning

#### 2.4.1. Construction of the Spreading Quality Model Based on TabPFN

**Figure 2:**
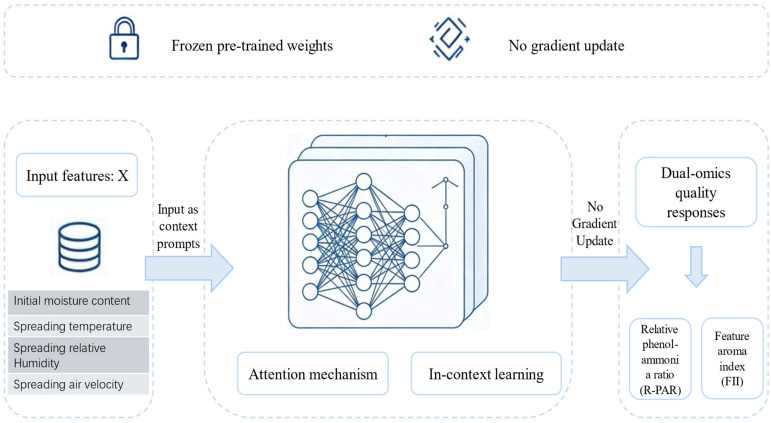
Construction workflow of the TabPFN model.

In this study, TabPFN was innovatively introduced as the core predictive model, with its construction workflow illustrated in Figure 2. By bypassing the cumbersome gradient updates and hyperparameter tuning processes characteristic of traditional algorithms, the model leverages the prior knowledge and in-context learning capabilities inherent in its meta-learning mechanism to achieve highly robust nonlinear Bayesian inference within a minimal sample space.

The input feature space (*X*) of the model was precisely constrained to four key processing parameters: initial moisture content, spreading temperature, relative humidity, and airflow velocity. The output response space (*Y*) was defined by dual-omic quality indicators: R-PAR and FII.

#### 2.4.2. Multi-Objective Synergistic Optimization of the Spreading Process Based on Taste and Aroma Quality

**Figure 3:**
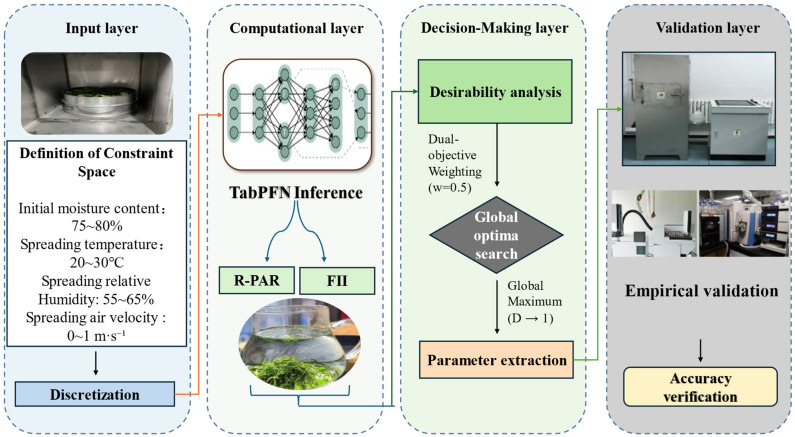
Flowchart of the multi-objective synergistic optimization of the green tea spreading process based on the TabPFN model.

Targeting the metabolic transformation and formation of taste (R-PAR) and aroma (FII), a multi-objective synergistic function was constructed. The optimization strategy, as illustrated in Figure 3, relied on the high-precision TabPFN model established previously (Section 2.4.1). Within the defined constraint space (initial moisture content of 75–80%, spreading temperature of 20–30 °C, spreading relative humidity of 55–65%, and spreading airflow velocity of 0–1 m·s^−1^), a high-resolution virtual process grid was generated using fixed step sizes. The virtual process grid data were inputted into the TabPFN to execute high-throughput inference, thereby reconstructing the global quality predictions.

To ensure the biochemical and physical validity of the data-driven model inferences, a mandatory boundary constraint was imposed prior to the multi-objective synergistic optimization, requiring the predicted output to satisfy R-PAR ≥ 0. This prevented the generation of physically meaningless solutions potentially caused by extreme model extrapolation. At the decision level, the Overall Desirability (D) function was introduced. Since a smaller R-PAR indicates superior quality, it was first subjected to reverse standardization; conversely, as a larger FII indicates better quality, it underwent forward standardization. Both objectives were assigned equal weights of 0.5 to calculate the comprehensive Dvalue for each virtual node. By retrieving the global maximum of the Dvalue (where approaches 1), the theoretical optimal process combination was inversely resolved, and its predictive accuracy was subsequently verified through physical validation experiments.

### 2.5. Verification of Model Generalization Capacity

Three mainstream methods were selected to simultaneously construct spreading prediction models as controls, including RSM [31], PLSR [32] and SVR [33]. All models were strictly subjected to unified input feature spaces and data partitioning constraints. Prior to modeling, Z-score standardization was applied to the continuous input variables to eliminate the influence of dimensional discrepancies on model parameter optimization. The Leave-One-Out Cross-Validation (LOOCV) strategy was employed to evaluate the generalization capacity of the models [34]. In each validation iteration, one independent sample was selected as the test set, while the remaining samples served as the training set; this process was iterated until the entire dataset was traversed. Considering the limited sample size in this study, the average predictive coefficient of determination R_pred_^2^ and the average root mean square error (RMSE) were adopted as the core evaluation metrics for model generalization performance to authentically characterize the models’ generalization capacities. Values of R_pred_^2^ approaching 1 and RMSE approaching 0 indicate higher predictive accuracy and generalization capacity of the model.

The comparative analysis based on model cross-validation reflects the inferential performance of the models across the process response surface. This approach is highly suitable for small-sample modeling scenarios like tea processing, which are characterized by both high process complexity and strong nonlinear biochemical properties [35].

## 3. Results

### 3.1. Mechanical Stress Effects of the Spreading Process on the Microstructure of Fresh Tea Leaves

**Figure 4:**
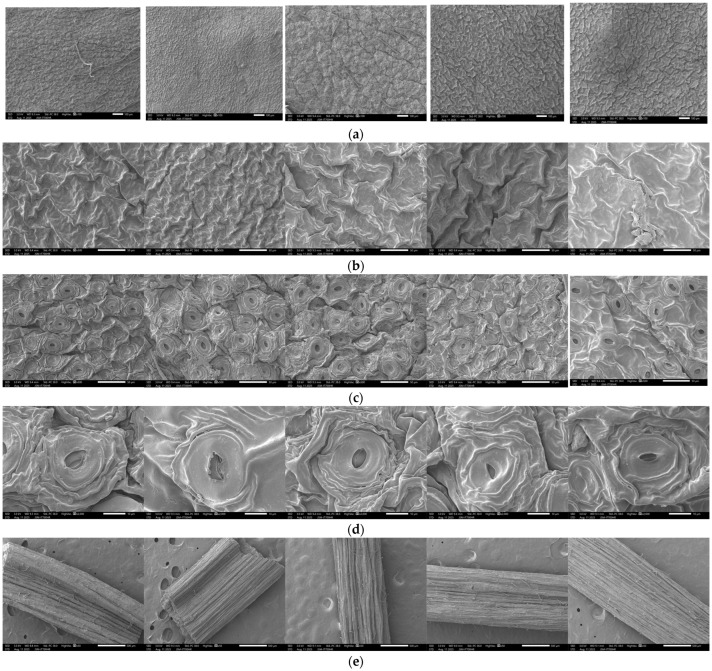

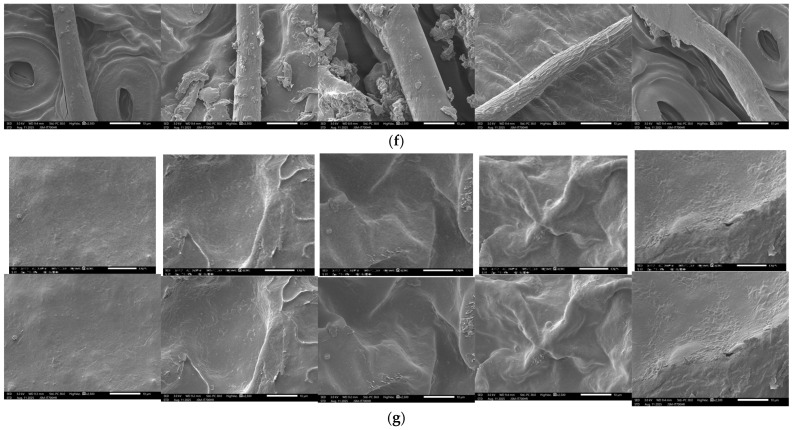
Multidimensional microstructural and mechanical stress evolution of fresh tea leaves at different stages of the spreading process: (**a**) overall structure of epidermal cells (100×); (**b**) microstructural evolution of mechanical epidermal shrinkage (500×); (**c**) distribution and initial state of stomata (500×); (**d**) high-magnification microscopic features of stress-induced stomatal closure (2000×); (**e**) contracted structure of vascular bundles in tea stalks (50×); (**f**) curling and structural damage characteristics of trichomes (2500×); (**g**) textural changes in cuticular wax and evolution of drought-induced microcracks (2500×). Note: Columns 1 to 5 correspond to Group 10, Group 4, Group 15, Group 16, and Group 9, respectively.

The leaf microstructures of all experimental groups were characterized via SEM. As illustrated in Figure 4, Groups 10, 4, 15, 16, and 9 were selected as representative samples for the following in-depth analysis.

In this study, representative experimental groups (Groups 10, 4, 15, 16, and 9) were selected for multi-scale characterization through scanning electron microscopy (SEM) observations, as illustrated in Figure 4. Group 10 (Figure 4, Column 1), treated under a higher initial moisture content of 80%, exhibited flattened epidermal cell morphology (Figure 4a), turgid guard cells with slightly open stomata (Figure 4d), and dense, intact cuticular wax textures (Figure 4g). These observations indicate relatively stable physical barriers and biochemical compartmentalization structures within the leaves under these processing conditions. Group 4 (Figure 4, Column 2) displayed initial wilting characteristics, with the epidermis beginning to show shallow shrinkage (Figure 4b), reflecting early physical deformation due to water loss. Group 15 (Figure 4, Column 3), although in a dehydrated state, exhibited no significant changes in the shriveled morphology of epidermal cells or stomatal status (Figure 4d), attributed to the process condition of 0 m/s wind speed, which resulted in a lack of convective drying dynamics. Group 16 (Figure 4, Column 4) demonstrated moderate stress characteristics under the coupling of moisture and convective drying. Microscopic morphology revealed distinct furrow-like shrinkage in epidermal cells (Figure 4b), with stomata semi-closed due to dehydration stress (Figure 4d). However, defensive structures such as cell edges and the cuticle (Figure 4g) maintained basic physical continuity without noticeable tearing. Notably, this moderate mechanical stress may lay the foundation for the subsequent transformation of flavor compounds and aromatic components. In contrast, Group 9 (Figure 4, Column 5) experienced severe dehydration stress. The microscopic features exhibited twisted and contracted vascular bundles in tea stems (Figure 4e) and wilted guard cells (Figure 4d). Additionally, distinct drought-induced cracks and localized physical tears on the cuticle surface were revealed under higher magnification (Figure 4g). These observations reflect the mechanical damage inflicted on fresh leaf tissues under extreme Spreading conditions.

It should be noted that the SEM observations described above focused on two-dimensional morphological changes in the leaf epidermal structure. The microscopic changes in mesophyll cells and their internal organelles (vacuoles, chloroplasts), which are the core sites for the transformation of taste and aroma compounds in tea, cannot be fully inferred from the degree of epidermal shrinkage alone. Therefore, the microstructure–quality index associations established in this study should be understood as indirect morphological evidence of spreading stress intensity, rather than a rigorous validation of causal mechanisms at the organelle level.

### 3.2. Results and Analysis of R-PAR Based on UHPLC-HRMS

#### 3.2.1. Global Metabolic Profiling and QC Results

To elucidate the remodeling mechanism of composite Spreading processes on tea metabolomics, this study conducted an in-depth analysis of experimental samples subjected to various Spreading processes using UHPLC-HRMS, coupled with multidimensional databases (NovoMetDB, HMDB, KEGG, LIPIDMAPS).

**Figure 5:**
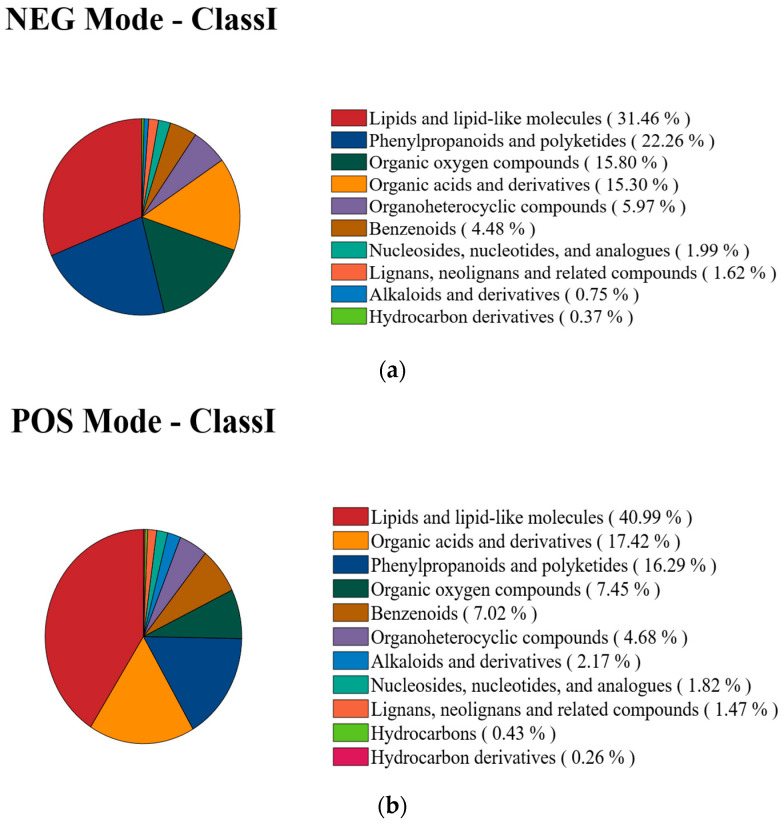
Primary Classification of Metabolites Identified by Non-Targeted Metabolomics During Green Tea Spreading. (**a**) Pie chart of primary metabolite classification identified in negative ion mode; (**b**) Pie chart of primary metabolite classification identified in positive ion mode.

Quality control (QC) results demonstrated that the Pearson correlation coefficients among all QC samples exceeded 0.99 in both positive and negative ion modes (Figure S1), with mass spectrometry mass errors strictly controlled within ≤5 ppm, thereby confirming high instrument analytical batch stability and data reliability. Based on the filtering mechanism, a total of 2473 metabolites were identified in both positive and negative ion modes. Based on the level of identification confidence, all metabolites were classified into three categories: standard-verified compounds (satisfying MS1, MS2, and retention time matching simultaneously), totaling 360; MS2-confirmed compounds (satisfying MS1 and MS2 matching), totaling 1144; and putatively annotated compounds (satisfying only MS1 matching), totaling 969. In addition, unstable feature peaks with CV > 30% in QC samples were strictly filtered out during the data preprocessing stage. Primary classification statistics (Figure 5a,b) revealed that the metabolites were highly enriched in categories such as phenylpropanoids, polyketides, organic acids and their derivatives, as well as lipids. These abundant polyphenols and amino acid groups constitute the foundational taste substances of green tea. The KEGG annotation results (Figure S2) indicated that metabolites exhibited the most significant enrichment signals in amino acid metabolism and carbohydrate metabolism, with a total of 62 amino acid-related metabolites annotated in both positive and negative modes, reflecting the strong influence of Spreading and dehydration on fundamental carbon and nitrogen metabolism in tea leaves. Meanwhile, the HMDB database annotation results (Figure S3) further revealed that metabolites were significantly clustered in phenylpropanoids and polyketides, which are key precursors for tea polyphenol synthesis. Additionally, the LIPIDMAPS database annotation results (Figure S4) indicated a significant accumulation of lipids and lipid-like molecules, suggesting the activation of pathways involved in membrane lipid degradation.

Based on comprehensive multi-dimensional annotation results, fresh tea leaves exhibit distinct coordinated responses in carbon and nitrogen metabolism during the Spreading process. Nitrogen metabolism, represented by free amino acid metabolism, evolves synchronously with carbon metabolism, characterized by phenylpropanoid and polyphenol synthesis. The dynamic transformation between these two pathways constitutes the core biochemical basis for the fluctuation in the taste quality of tea soup, providing robust metabolomic evidence for the subsequent construction and optimization of R-PAR.

#### 3.2.2. Screening of Core Taste-Active Compounds and Construction of R-PAR

The screening of core taste-active compounds followed a rigorous stepwise procedure to ensure data reliability and sensory relevance. According to the quality control standards for metabolomic data, unstable feature peaks with a coefficient of variation (CV) greater than 30% in all quality control (QC) samples were first eliminated to remove instrumental noise and false-positive interferences from unstable detection. Subsequently, unsupervised principal component analysis (PCA) was performed on the globally QC-cleaned UHPLC-HRMS profiles, and a preliminary set of candidate compounds highly responsive to spreading stress was identified using an absolute eigenvector loading threshold of |Loadings| > 0.05. This candidate pool was then refined using established knowledge of taste contribution [36] and the criterion of relative abundance dominance, further eliminating interfering substances with strong mass spectrometric response but low sensory activity. The final selection was narrowed to the core constituents governing the astringency–umami balance after spreading: among polyphenols, six flavonoid glycosides and three catechins known to drive the bitter–astringent evolution of green tea were selected; among amino acids, L-glutamic acid, L-theanine, and L-aspartic acid were chosen (detailed information on the core compounds is provided in Table 2). In terms of compound identification, the mass errors of all targeted markers were strictly controlled within ≤5 ppm, and accurate matching was achieved by integrating the NovoMetDB database and a high-quality local MS2 spectral library. After the above process, the 12 core substances shown in Table 2 were finally established as the chemical basis for defining the R-PAR. This selection aligns with the consensus of previous metabolomic studies on tea processing [37].

**Figure 6:**
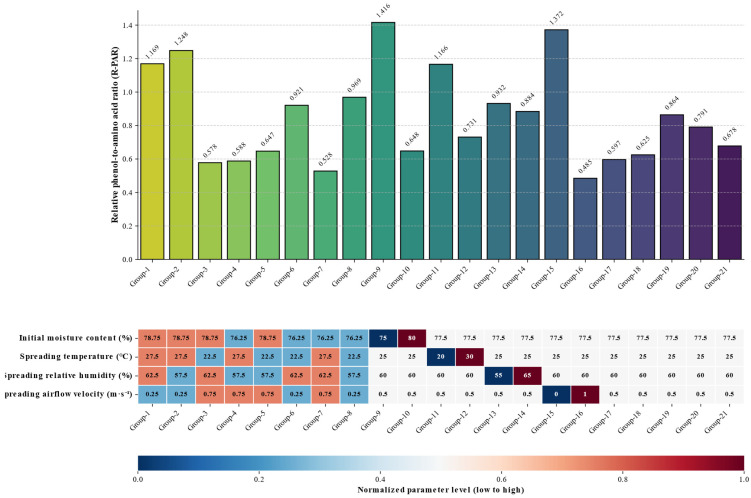
Correlation map between the Spreading process and R-PAR.

Based on the twelve core taste substances ultimately identified, their corresponding chromatographic peak areas were extracted and subjected to range normalization. The relative phenolic-to-amino ratios for the various processing groups were calculated, as illustrated in Figure 6. This dimensionless characteristic index was established to characterize the evolution of core taste substances under different spreading and Spreading processes.

### 3.3. Results and Analysis of Characteristic Aroma Index Based on HS-SPME-GC-MS

#### 3.3.1. Volatile Metabolite Profiling and Metabolic Pathway Analysis

**Figure 7:**
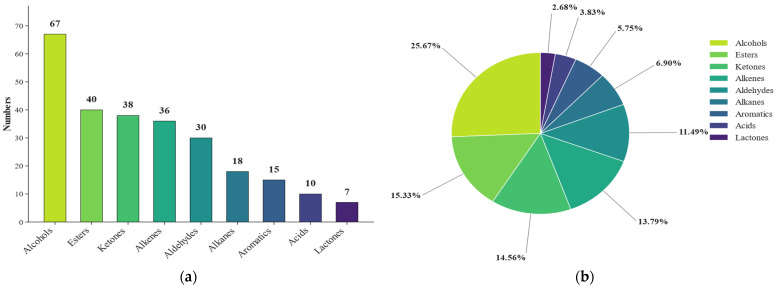
Number and proportion of different aroma categories in green tea: (**a**) number of volatile compounds; (**b**) relative proportion.

After chromatographic peak deconvolution and matching, along with rigorous denoising pretreatment (eliminating derivatization mismatches and trace environmental contaminants), a total of 261 volatile compounds were identified in the test samples (Table S1). Detailed classification and retention indices (RI) of all volatile constituents are provided in Table S1. Based on their chemical structures, these compounds were categorized into nine classes (Figure 7a). Among them, alcohols were the most abundant (67 compounds, 25.67%), followed by esters (40, 15.33%), ketones (38, 14.56%), alkenes (36, 13.79%), and aldehydes (30, 11.49%), together accounting for over 80% of the total. Alkanes (18, 6.90%) and aromatic compounds (15, 5.75%) each exceeded ten compounds, whereas acids (10, 3.83%) and lactones (7, 2.68%) constituted relatively low proportions in terms of compound numbers (Figure 7b). Consistent with previous findings [38], esters, alcohols, and ketones were the predominant aroma constituents of steamed green tea, further confirming the reliability of the analytical results.

**Figure 8:**
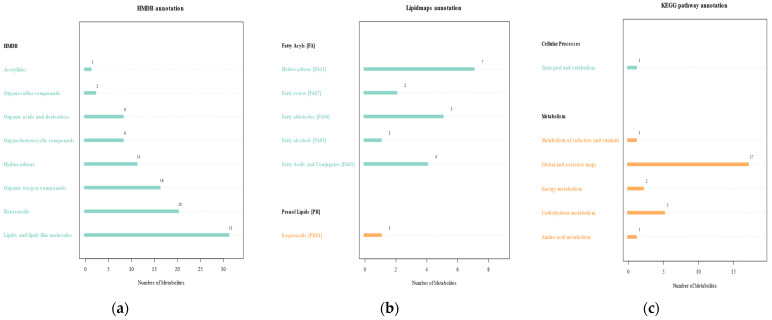
Biological pathway annotation and functional classification of volatile metabolites in green tea: (**a**) HMDB superclass classification; (**b**) LIPID MAPS lipid classification; (**c**) KEGG pathway annotation.

To investigate the formation mechanism of aroma under the physical stress of spreading and Spreading, the metabolites detected by HS-SPME-GC-MS were mapped to the HMDB, KEGG, and LIPIDMAPS databases (Figure 8). Cross-annotation between HMDB (Figure 8a) and LIPIDMAPS (Figure 8b) revealed a predominance of lipids and lipid-like molecules. This finding confirms that as water loss occurs during the processes of spreading and Spreading, the metabolic pathways of hydrolytic enzymes are significantly activated, promoting the release of fatty aldehydes and alcohols, which form an essential foundation for the aroma profile of the spread-withered leaves. Furthermore, the accumulation of other core aroma compounds follows distinct pathway branches. The isoprenoids detected in LIPIDMAPS and the phenolic compounds in HMDB constitute the critical structural backbone of characteristic aroma components. The KEGG enrichment analysis (Figure 8c) further indicates that aromatic compounds are highly active in carbohydrate and amino acid metabolic pathways. This suggests that during the spreading process, not only does membrane lipid degradation occur, but the enzymatic release of glycosidic aroma precursors and the transformation of free amino acids also undergo intense and synchronous evolution, thereby laying the foundation for the accumulation of compounds such as terpenoid alcohols (linalool) and aromatic alcohols (benzyl alcohol). These findings are consistent with previous research conclusions [39]. In summary, the aroma evolution of fresh tea leaves during the spreading process represents a dynamic balance driven by both lipid degradation metabolism and isoprenoid/aromatic metabolism. This principle provides theoretical support for subsequent calculations of core substance ROAV and the construction of FII.

#### 3.3.2. Screening of Odor-Active Compounds and FII Construction

To quantitatively assess the evolution of dominant aroma compounds during the green tea spreading process, this study performed preliminary screening based on volatile metabolomics profiles, utilizing a threshold of relative odor activity value ROAV ≥ 0.1. Fourteen characteristic aroma compounds, which are likely to significantly contribute to the overall aroma profile, were identified, as detailed in Table 3 below.

Subsequently, a core threshold of ROAV ≥ 1 was applied to the candidate substances to identify the odor-active compounds dominating the aroma profile of each sample. The results revealed that across all test samples, only β-ionone (ROAV range 12.19–100) and linalool (ROAV range 4.41–24.66) consistently and stably exhibited ROAV values greater than 1, and together they accounted for over 98% of the total ROAV of all floral-type compounds, establishing them as the dominant contributors to the characteristic floral aroma of green tea after spreading. In addition, nonanal, 1-octen-3-ol, and geraniol also attained ROAV ≥ 1 in certain treatment groups, indicating that spreading exerts a certain shaping effect on non-floral aroma profiles as well. The ROAV values of other substances such as hexanal and (Z)-3-hexen-1-ol were all below 1, playing only a modifying role and were therefore not included in the FII calculation. This result aligns closely with the findings of Gui et al. [40] regarding the evolution of aroma compounds during green tea processing. In terms of compound identification, all volatile components were subjected to dual qualitative confirmation through mass spectral similarity matching and retention index (RI) matching against the NIST 2020 database.

**Figure 9:**
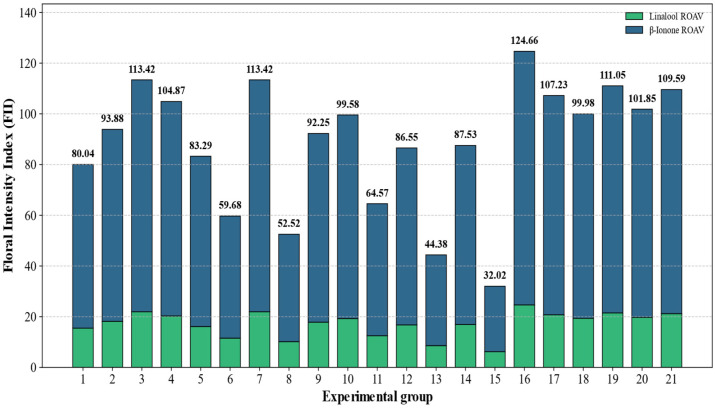
FII of each experimental group and the contribution of individual components within each group.

Given the absolute dominance of β-ionone and linalool in aroma contribution (accounting for >98%), the Floral Intensity Index (FII) of green tea was defined as the sum of the ROAV values of β-ionone and linalool. The FII values of each sample are illustrated in Figure 9, ranging from 32.02 to 124.66, indicating that this index exhibits extremely high sensitivity to variations in the Spreading process. The establishment of the FII facilitates objective quantitative characterization of the evolution of characteristic aroma quality.

### 3.4. Quality Prediction, Multi-Objective Process Optimization Based on TabPFN

#### 3.4.1. Evaluation of the Tea Spreading Quality Prediction Model

Building on the TabPFN training methodology established earlier, this section independently evaluates the model’s predictions for two quality indicators of green tea spreading. TabPFN successfully captured the nonlinear regulatory effects of varying spreading conditions on both core polyphenols and key aroma compounds. The input and output variables of the model are defined as shown in Table 4 below. Specifically, the taste proxy indicator (R-PAR) in the output was calculated based on the 12 core metabolites described in Table 2 (6 flavonoid glycosides, 3 catechins, and 3 amino acids); the Floral Intensity Index (FII) was derived by summing the relative odor activity values (ROAV) of the two core floral volatile compounds (β-ionone and linalool) listed in Table 3.

In terms of predictive performance, the model achieved an R_pred_^2^ of 0.81 and an RMSE of just 0.11 for R-PAR; for FII, it attained an R_pred_^2^ of 0.77 and an RMSE of 13.72. Notably, given the inherently high volatility of tea aroma compounds driven by environmental and biological variability, the RMSE of 13.72 for FII falls well within the accepted tolerance band for digital modeling in food processing.

Overall, these results demonstrate that the TabPFN model can effectively learn the mapping between spreading conditions and the core taste and aroma components, with an inference accuracy that meets current analytical needs. This provides a viable machine-learning foundation for the subsequent global multi-objective optimization.

#### 3.4.2. Multi-Objective Synergistic Optimization Results

To identify the spreading parameter combination that delivers the optimal balance between taste and aroma, a grid-based virtual optimization was carried out using the TabPFN model. The search revealed that as the D-value approached its optimum, the model identified an optimal set of process conditions: initial moisture content 76.8%, spreading temperature 26.2 °C, relative humidity 61.5%, and airflow rate 0.85 m·s^−1^. Under these conditions, the predicted R-PAR dropped to 0.465, while the FII climbed to 125.70. The 95% confidence intervals of the predicted means at the optimal point were: R-PAR [0.41, 0.52] and FII [119.3, 132.1].

To verify the reliability of the model predictions, fresh leaves of Echa No. 10 from the same season were used, and three independent, complete spreading and detection runs were performed as parallel validation experiments based on the practically achievable process parameters: initial moisture content 77.0%, spreading temperature 26.0 °C, relative humidity 61.5%, and airflow rate 0.9 m·s^−1^. The comparative results are summarized in Table 5. The measured R-PAR averaged 0.478 ± 0.015, and the FII averaged 122.98 ± 3.26. The intra-group relative standard deviations (RSD) were 3.14% and 2.65%, respectively, both well below the commonly accepted 5% quality-control threshold for tea physicochemical testing, indicating satisfactory repeatability and reliable measurements. All model-predicted values fell completely within the 95% confidence intervals of the measured values, and the confidence intervals of the predicted means overlapped with those of the measured values, indicating no statistically significant difference between the predicted and measured values. This confirms the accuracy and reliability of the TabPFN model in predicting the dual quality indicators under the optimal spreading process.

As corroborated by prior research, micro-perturbations in processing parameters exert a decisive influence on the characteristic aroma quality of tea [41]. In this study, the optimal spreading configuration identified by the model validates the accuracy of TabPFN in characterizing the complex nonlinear relationship between the spreading process and quality attributes. Furthermore, these findings substantiate the immense potential of process optimization for the targeted modulation of taste and aroma.

### 3.5. Model Validation and Mechanistic Analysis

#### 3.5.1. Model Performance Comparison

**Figure 10:**
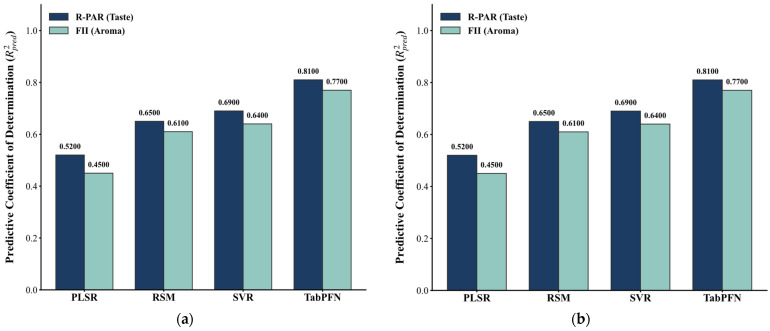
Comprehensive evaluation and benchmarking of different prediction models: (**a**) comparison of prediction R_pred_^2^ for FII and R-PAR across four models; (**b**) heatmap visualization of global prediction performance covering R_pred_^2^ and root mean square error (RMSE). Color saturation encodes the performance level, with dark blue representing higher accuracy and lower error.

Table 6 compares the performance of TabPFN with three widely used models: response surface methodology (RSM), partial least squares regression (PLSR), and support vector regression (SVR). The results show that TabPFN exhibited superior generalization for both quality indicators, as visualized in Figure 10.

In the prediction of R-PAR, TabPFN achieved a predictive coefficient of determination (R_pred_^2^) of 0.81 and a root mean square error (RMSE) of 0.11. Compared to the second-best performing SVR model (R_pred_^2^ = 0.69, RMSE = 0.142), TabPFN demonstrated a 17.4% relative increase in R_pred_^2^ and a 27.6% reduction in RMSE. Regarding FII prediction, the TabPFN model reached an R_pred_^2^ of 0.77 and an RMSE of 13.72, representing improvements of 20.3% and 20.1%, respectively, over SVR (R_pred_^2^ = 0.64, RMSE = 17.17). The above results indicate that the TabPFN model achieved a favorable level of performance in the small-sample food processing modeling scenario, but has not yet reached the threshold of excellence. This performance level demonstrates that the current model can effectively capture the major variation trends between process parameters and quality indicators, yet approximately 19–23% of the variation remains unexplained. This unexplained variation may originate from biological fluctuations among fresh leaf batches, inherent detection errors in non-targeted metabolomic data, and potential influencing factors not included in the input space. Therefore, the results of this study should be positioned as a proof-of-concept validation of TabPFN in this application scenario, rather than a mature model meeting industrial-grade precision requirements.

#### 3.5.2. Mechanistic Interpretation of Performance Discrepancies and Application Suitability

**Figure 11:**
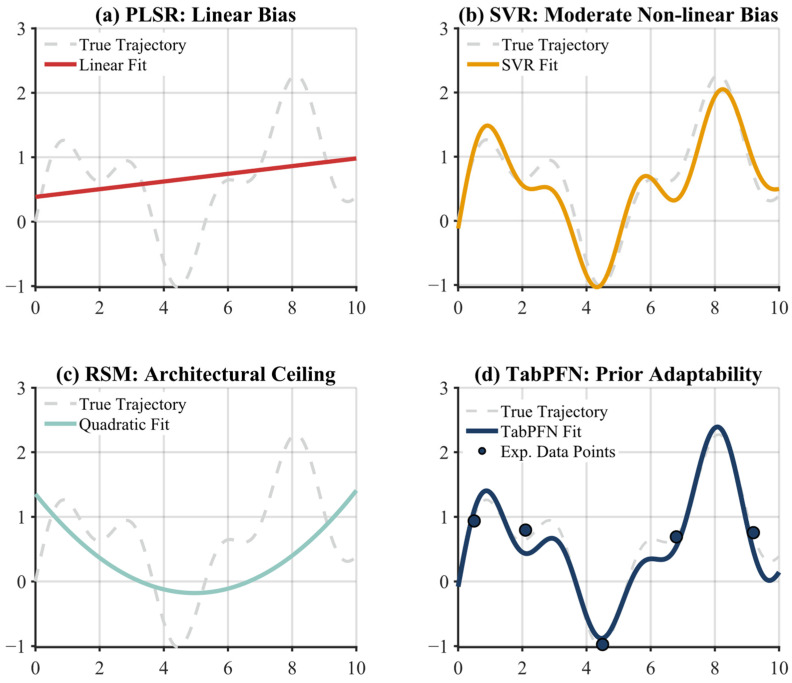
Schematic comparison of inference mechanisms among different prediction architectures during tea spreading: (**a**) partial least squares regression (PLSR); (**b**) support vector regression (SVR); (**c**) response surface methodology (RSM); (**d**) tabular prior-data fitted network (TabPFN).

Viewed through the lens of model principles and their adaptability to green tea spreading scenarios, a comparison of the inference mechanisms underlying the various modeling strategies is illustrated in Figure 11.

When addressing such small-sample process modeling scenarios, the three mainstream modeling approaches exhibit certain inherent limitations. Although RSM provides acceptable macroscopic predictive capability within the experimental design range, it is constrained by the fixed structure of second-order polynomials, which fails to precisely characterize the higher-order nonlinear regulatory effects of processing parameters on the metabolism of taste and aroma compounds. The linear modeling assumption of PLSR struggles to adequately capture the complex nonlinear associations between processing conditions and the metabolism of polyphenols and amino acids. Regarding SVR, kernel extrapolation in the sparse regions of small samples is dominated by a limited number of support vectors, thereby lacking physical constraints consistent with enzymatic kinetics and continuous water loss patterns.

In contrast, TabPFN compensates for these core deficiencies. By performing meta-learning on massive synthetic tabular datasets, universal statistical priors are solidified into the network parameters. This allows the model to construct smooth and robust nonlinear mappings within a sparse process parameter space without requiring gradient iteration or hyperparameter tuning on the small sample set, effectively mitigating the risks of overfitting and multicollinearity. This not only elucidates the superior performance demonstrated in Table 5 but also substantiates that the optimal spreading process derived from this model is both highly scientific and reliable.

### 3.6. Research Limitations

This study, by integrating multi-dimensional omics techniques with a small-sample machine learning model, provides an effective pathway for the intelligent optimization of the green tea spreading process. However, to comprehensively evaluate the scientific boundaries and engineering application potential of this study, the following limitations require systematic and critical discussion:

(1) Limitations of proxy indicators and analytical sample sensory validation. This study focused on elucidating the quality evolution during the single spreading process; therefore, microwave fixation was directly applied to terminate the experiment once spreading was completed. From a processing perspective, the tea leaves did not undergo the complete manufacturing process, and consequently, no quantitative professional sensory descriptive analysis (QDA) validation was conducted.
(2) The model development data were derived solely from spring fresh leaves of a single tea cultivar (Echa No. 10), and the model has not yet been validated on independent external datasets. In actual agricultural production, the metabolism of fresh tea leaves fluctuates with cultivar genotype and environmental factors across harvesting seasons. Although TabPFN has demonstrated inferential robustness on small samples, this scale remains insufficient to cover the extreme environmental conditions and fresh leaf moisture states encountered in natural settings.
(3) At the level of industrial application, this study was conducted on a multi-parameter controllable thin-layer test bench. In large-scale industrial continuous production, unevenness in tea leaf pile thickness and non-steady-state variations in workshop temperature and humidity will introduce substantial environmental noise. The current model remains at the proof-of-concept stage. Before formal deployment in large-scale production, it is imperative to establish an expanded database covering multiple cultivars and multiple seasons, and to incorporate online monitoring data from actual production lines for dynamic model calibration and iterative updating.

## 4. Conclusions

This study systematically elucidated the microstructural alterations and the dynamic trajectories of taste and aroma profiles under water-deficit stress during green tea spreading. Furthermore, a high-fidelity predictive model for dual-quality indicators, specifically optimized for small-sample scenarios, was successfully established. The core conclusions are as follows:

(1) SEM observations confirmed that water loss during spreading disrupts the biochemical compartmentalization within fresh leaf cells, thereby establishing the requisite biophysical environment for subsequent enzymatic transformations. Additionally, metabolomic-driven approaches were employed to precisely quantify R-PAR and FII.
(2) Addressing the inherent challenge of small sample sizes in tea processing research, this study employed the TabPFN algorithm to construct a high-precision quality prediction model. The synergistic optimization of taste and aroma was achieved under the following optimal parameters: an initial moisture content of 76.8%, a spreading temperature of 26.2 °C, a spreading relative humidity of 61.5%, and a spreading airflow velocity of 0.85 m·s^−1^. Under these optimal conditions, the R-PAR and FII reached 0.465 and 125.70, respectively.
(3) The predictive accuracy and generalization capacity of the TabPFN model significantly outperformed both traditional baseline models (RSM and PLSR) and conventional machine learning algorithms (SVR). This substantiates the scientific reliability of utilizing TabPFN for quality prediction in the green tea spreading process.
(4) The model in this study was constructed based on spring fresh leaves of a single tea cultivar, and its cross-cultivar and cross-season generalization ability remains to be further validated. R-PAR and FII are chemical proxy indicators, and their quantitative relationship with human sensory evaluation results still requires subsequent investigation to support the industrial application of the model.

## Figures and Tables

**Table 1:** Experimental factors and levels of the fresh tea leaf spreading process.

Level	Initial Moisture Content/%	Spreading Temperature/°C	Spreading Relative Humidity/%	Spreading Airflow Velocity/(m·s^−1^)
1	75.00	20.00	55.00	0.00
2	76.25	22.50	57.50	0.25
3	77.50	25.00	60.00	0.50
4	78.75	27.50	62.50	0.75
5	80.00	30.00	65.00	1.00

**Table 2:** Chromatographic and mass spectrometric identification data for the core targeted markers.

No.	Name	Formula	Molecular Weight	*m*/*z*	RT (min)
1	Quercetin 3-O-rutinoside	C_27_H_30_O_16_	610.15339	609.14683	5.441
2	Quercetin 3-O-galactoside	C_21_H_20_O_12_	464.09548	463.08867	5.467
3	Kaempferol 3-O-rutinoside	C_27_H_30_O_15_	594.15848	593.15188	5.544
4	Kaempferol 3-O-glucoside	C_21_H_20_O_11_	448.10056	449.10744	5.561
5	Myricetin 3-O-galactoside	C_21_H_20_O_13_	480.0904	479.08365	4.819
6	Kaempferol 3-O-galactoside	C_21_H_20_O_11_	448.10056	447.0937	5.568
7	Epigallocatechin gallate	C_22_H_18_O_11_	458.08492	457.07767	5.129
8	(-)-Epicatechin gallate	C_22_H_18_O_10_	442.09	441.08299	6.455
9	(-)-Epigallocatechin	C_15_H_14_O_7_	306.07396	305.06642	4.998
10	L-Glutamic acid	C_5_H_9_NO_4_	147.05316	148.06026	1.496
11	L-Theanine	C_7_H_14_N_2_O_3_	174.10044	175.10716	1.909
12	L-Aspartic acid	C_4_H_7_NO_4_	133.03751	132.0305	1.249

**Table 3:** High-contribution candidate aroma compounds in green tea.

IUPAC Name	RT [min]	Avg Calculated RI	Library RI	Concentration Range (μg/kg)
Linalool	13.703	1100	1082	135.42–756.94
Geraniol	16.58	1258	1273	24.71–705.68
Benzyl alcohol	12.191	1042	1036	15.31–997.28
Benzeneacetaldehyde	12.374	1049	1081	23.82–135.01
β-Ionone	19.899	1493	1457	18.56–215.40
Acetophenone	12.978	1072	1029	1.48–20.91
(E)-Nerolidol	20.819	1568	1564	2.54–24.66
(Z)-Hex-3-en-1-yl hexanoate	18.439	1382	1389	11.27–50.64
Hexanal	5.029	804	806	2.04–356.46
(Z)-Hex-3-en-1-ol	6.637	857	868	2.04–339.61
D-Limonene	11.921	1031	1018	29.77–140.05
Nonanal	13.803	1105	1104	50.57–423.54
1-Octen-3-ol	10.516	981	969	19.66–352.47
1-Hexanol	7.041	871	860	0.23–371.68

**Table 4:** Summary of input and output variables for the TabPFN prediction model.

Variable Type	Variable Name	Unit/Range	Description
Input variables (Process parameters)			
X_1_	Initial moisture content	%	Moisture content of fresh leaves
X_2_	Spreading temperature	°C	Air temperature
X_3_	Spreading relative humidity	%	Air relative humidity
X_4_	Spreading airflow velocity	m·s^−1^	Forced convection air speed
Output variables (Quality indicators)			
Y_1_	R-PAR	Dimensionless	Taste proxy indicator
Y_2_	FII	Dimensionless	Aroma proxy indicator

**Table 5:** Comparison of model-predicted and experimentally measured values for the optimal spreading process parameters.

Quality Index	Predicted Value	95% CI of Predicted Mean	Experimental Value	RSD%	95% CI of Experimental Value
R-PAR	0.465	[0.41, 0.52]	0.478 ± 0.015	3.14%	[0.441, 0.515]
FII	125.70	[119.3, 132.1]	122.98 ± 3.26	2.65%	[114.88, 131.08]

**Table 6:** Comparison of predictive performance among different models for the dual quality indicators of the tea spreading process.

Model Type	Quality Index	R_pred_^2^	RMSE
PLSR	R-PAR	0.52	0.19
	FII	0.45	21.22
RSM	R-PAR	0.65	0.162
	FII	0.61	17.87
SVR	R-PAR	0.69	0.142
	FII	0.64	17.17
TabPFN	R-PAR	0.81	0.11
	FII	0.77	13.72

## Data Availability

The data presented in this study are available on request from the corresponding author.

## Supplementary Materials

The following supporting information can be downloaded at: https://www.mdpi.com/article/10.3390/foods15122069/s1, Figure S1: Pearson correlation of QC samples in positive and negative ion modes; Figure S2: KEGG pathway enrichment analysis; Figure S3: HMDB superclass classification of metabolites; Figure S4: LIPID MAPS classification of metabolites; Table S1: Detailed classification and retention indices of all volatile components.

## Abbreviations

The following abbreviations are used in this manuscript:

CCD	Central Composite Design
CV	Coefficient of Variation
DDA	Data-Dependent Acquisition
FII	Floral Intensity Index
GC-MS	Gas Chromatography–Mass Spectrometry
HMDB	Human Metabolome Database
HPLC	High-Performance Liquid Chromatography
HS-SPME	Headspace Solid-Phase Microextraction
KEGG	Kyoto Encyclopedia of Genes and Genomes
LIPID MAPS	Lipid Metabolites and Pathways Strategy
LOOCV	Leave-One-Out Cross-Validation
MS	Mass Spectrometry
PCA	Principal Component Analysis
PLSR	Partial Least Squares Regression
QC	Quality Control
RI	Retention Index
RMSE	Root Mean Square Error
ROAV	Relative Odor Activity Value
R-PAR	Relative Polyphenol-to-Amino Acid Ratio
RSD	Relative Standard Deviation
RSM	Response Surface Methodology
SEM	Scanning Electron Microscopy
SVR	Support Vector Regression
TabPFN	Tabular Prior-Data Fitted Network
UHPLC	Ultra-High Performance Liquid Chromatography
UHPLC-HRMS	Ultra-High Performance Liquid Chromatography–High-Resolution Mass Spectrometry
