# Supplementary material for: Predicting and Co-Optimizing the Taste and Aroma of Green Tea During Spreading Using the TabPFN Model
Source: Foods. 2026 Jun 8;15(12):2069. doi: 10.3390/foods15122069 (PMC13298301; doi:10.3390/foods15122069)
Supplement: Supplementary file 1 [file foods-15-02069-s001.zip › foods-4310202-supplementary.pdf]

Figure S1: Pearson correlation of QC samples in positive and negative ion modes;

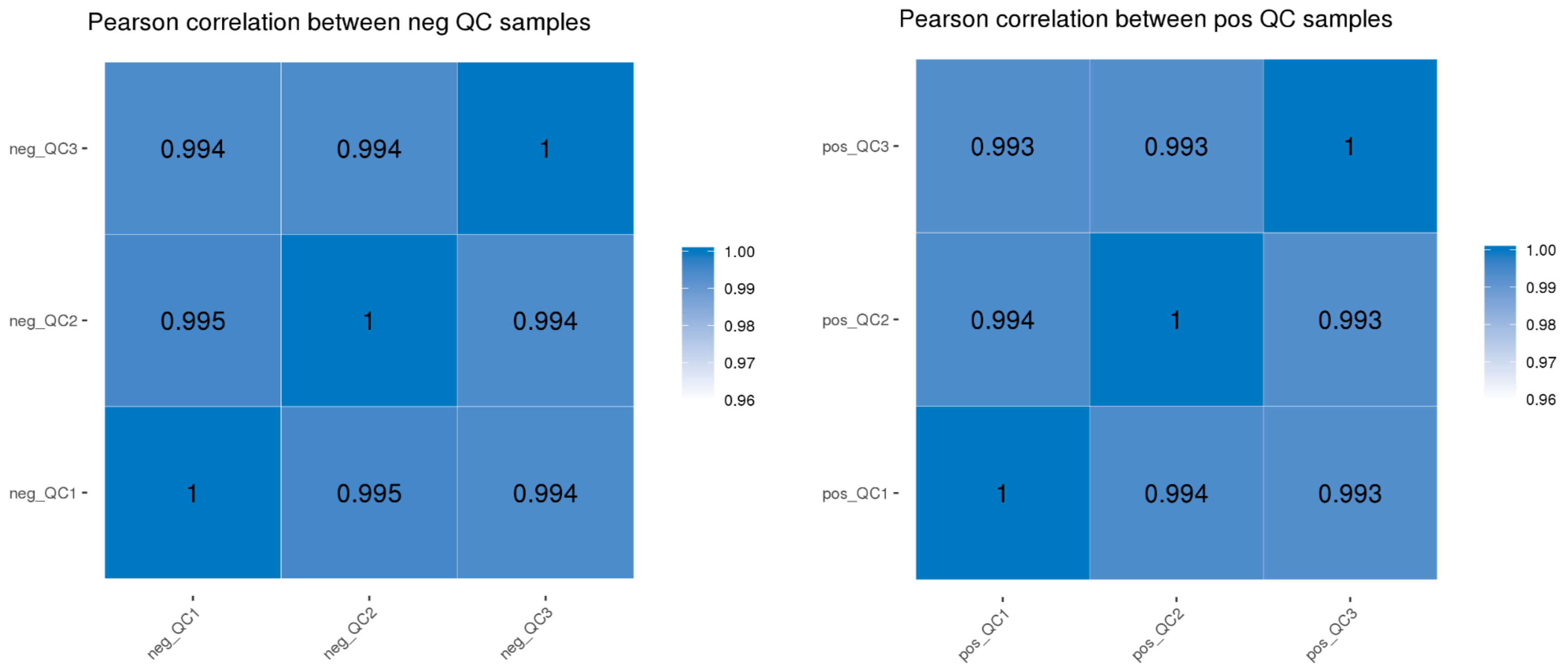

Figure S2: KEGG pathway enrichment anal-ysis;

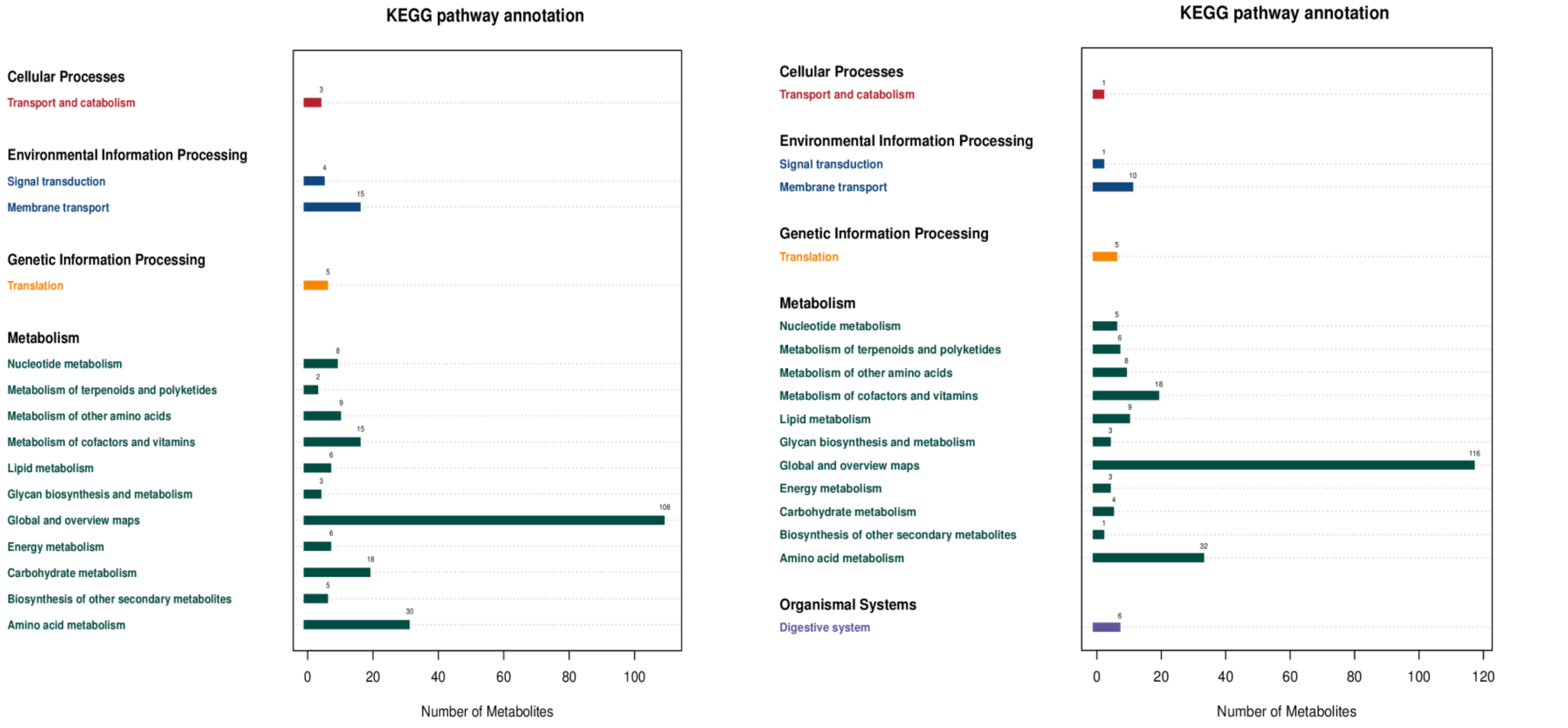

Figure S3: HMDB superclass classification of metabolites;

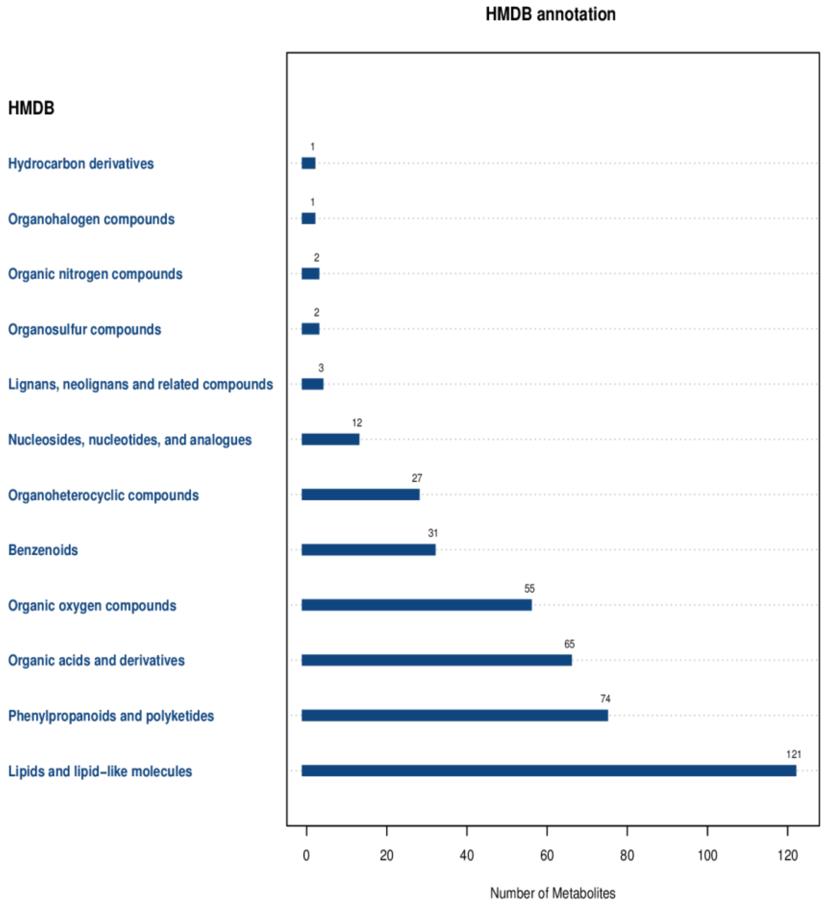

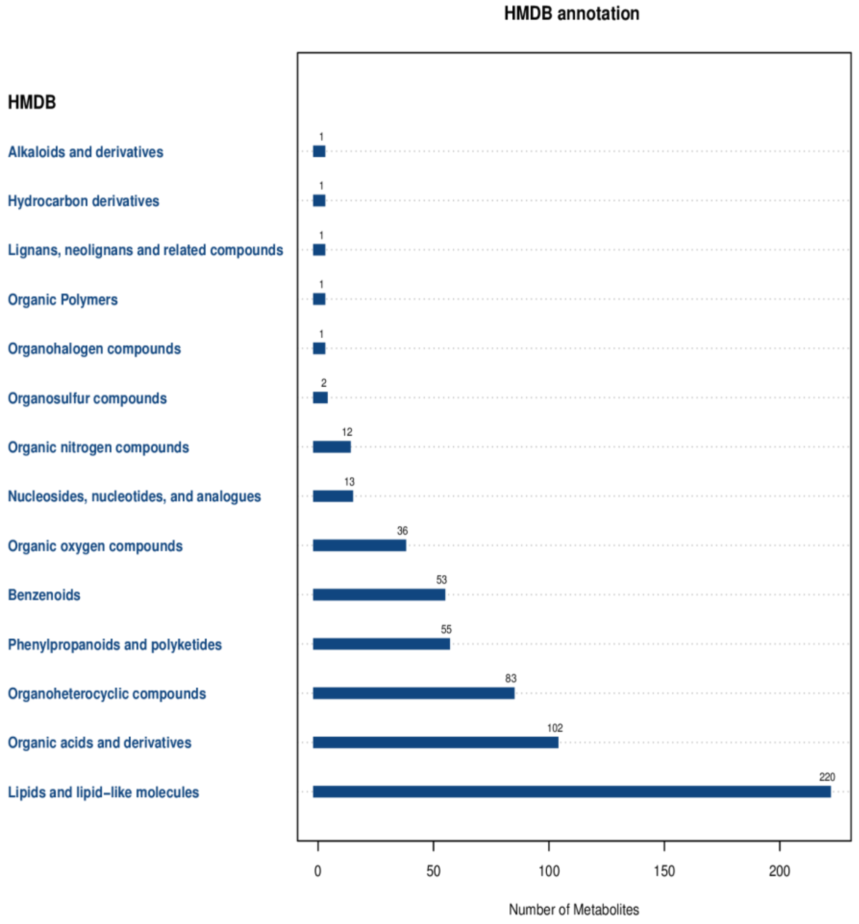

Figure S4: LIPID MAPS classifica-tion of metabolites;

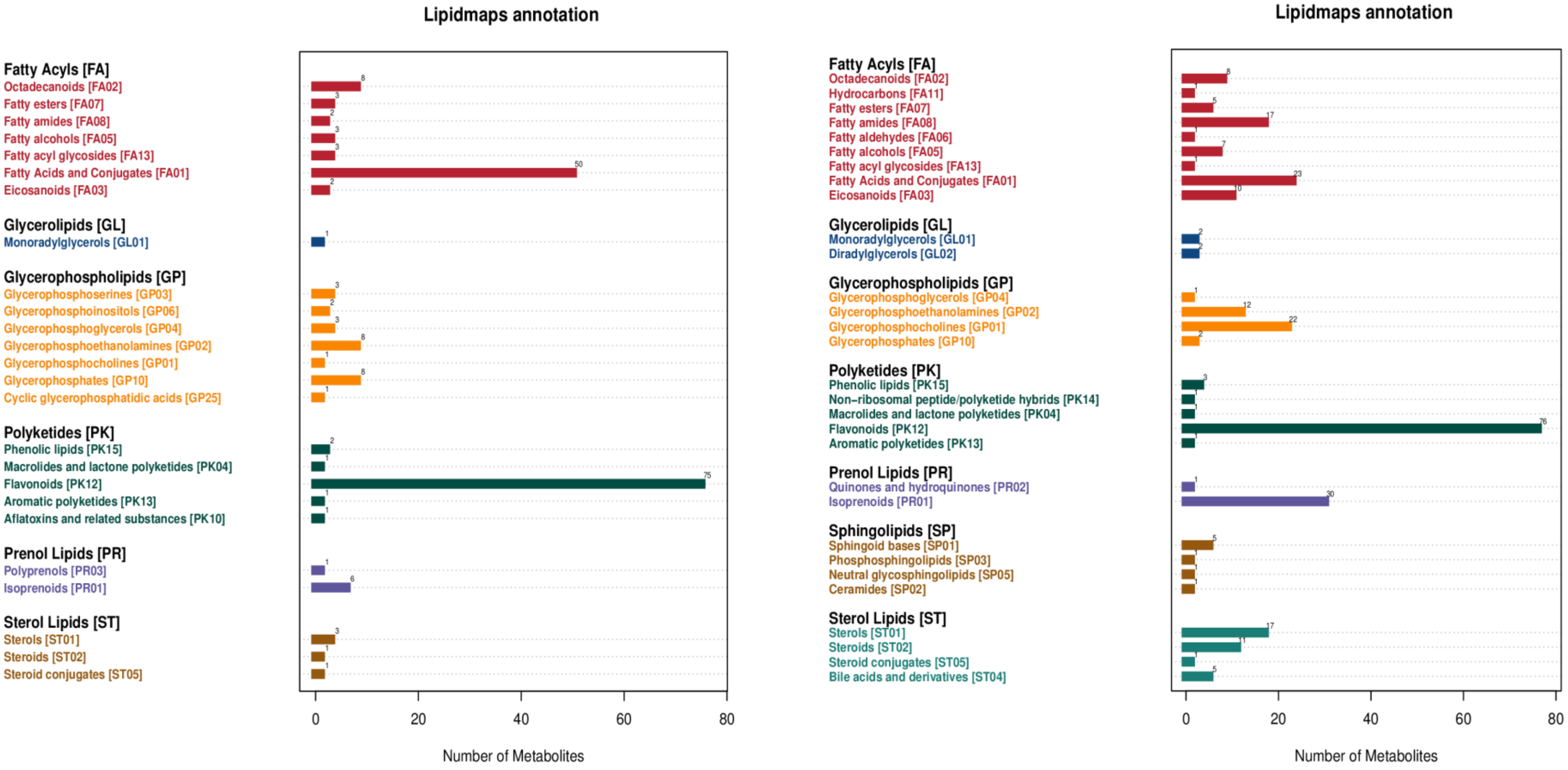

Table S1: Detailed classification and retention indices of all volatile compo-nents.

Compounds	Calculated RI	Identification Method
3-Methyl-2-butanone	599	RI,MS
Butanal	616	RI,MS
Methyl propanoate	653	RI,MS
3-Methylcyclopentene	659	RI,MS
Acetic acid	660	RI,MS
3-Methylbutanal	669	RI,MS
2-Methylbutanal	677	RI,MS
3-Pentanone	680	RI,MS
1-Penten-3-ol	695	RI,MS
1-Hydroxy-2-propanone	704	RI,MS
Pentanal	707	RI,MS
2-Ethylfuran	709	RI,MS
4-Pentyn-2-ol	713	RI,MS
Methyl isovalerate	724	RI,MS
Propanoic acid	724	RI,MS
Methyl 3-butenoate	728	RI,MS
3-Methyl-1-butanol	739	RI,MS
3-Ethyl-2,2-dimethylpentane	742	RI,MS
3-Methyl-1,4-heptadiene	742	RI,MS
2-Methylpropanoic acid	764	RI,MS
2-Methyl-4-pentenal	766	RI,MS
Ethyl hydroxyacetate	769	RI,MS
1-Pentanol	770	RI,MS

Tetrahydro-2-furanol	770	RI,MS
Cyclobutanemethanol	774	RI,MS
3-Isopropylcyclopentene	779	RI,MS
2-Hexanol	797	RI,MS
3-Methyl-2-hexanol	799	RI,MS
3-Hexen-2-one	804	RI,MS
Hexanal	804	RI,MS
Lactic acid	806	RI,MS
Dihydro-2-methyl-3(2H)-furanone	812	RI,MS
Methyl pentanoate	827	RI,MS
2,4-Dimethyl-1-heptene	842	RI,MS
2-Ethyl-5,5-dimethyl-1,3-cyclopentadiene	843	RI,MS
5-tert-Butyl-1,3-cyclopentadiene	846	RI,MS
1-(3-Ethylcyclobutyl)ethanone	847	RI,MS
3-Methylbutanoic acid	851	RI,MS
(Z)-3-Hexenal	855	RI,MS
(Z)-3-Hexen-1-ol	857	RI,MS
3-Hexen-1-ol	859	RI,MS
(Z)-4-Hexen-1-ol	860	RI,MS
2-Methylbutanoic acid	869	RI,MS
(E)-2-Hexen-1-ol	870	RI,MS
1-Hexanol	871	RI,MS
3-Ethylidene-2-methyl-1-hexen-4-yne	889	RI,MS
2-Heptanone	894	RI,MS
Heptanal	903	RI,MS
Pentanoic acid	908	RI,MS

Butyrolactone	917	RI,MS
β-Pinene	927	RI,MS
Methyl hexanoate	927	RI,MS
trans-β-Ocimene	932	RI,MS
Methyl (Z)-3-hexenoate	935	RI,MS
2,7-Dimethyloxepine	936	RI,MS
3-Isopropyl-2-cyclopenten-1-one	946	RI,MS
Ethyl 2-methyl-3-oxobutanoate	946	RI,MS
Camphene	948	RI,MS
1,2,3,3-Tetramethyl-4-methylenecyclopentene	948	RI,MS
Propylbenzene	953	RI,MS
1-tert-Butyl-4-methylcyclohexane	957	RI,MS
6-Methyl-2-heptanone	957	RI,MS
Cyclooctane	960	RI,MS
Benzaldehyde	961	RI,MS
Methyl 2-hexenoate	968	RI,MS
3-Methylnonane	971	RI,MS
3,7-Dimethyl-1,3,7-octatriene	973	RI,MS
1,7-Octadien-3-ol	976	RI,MS
1-Octen-3-ol	981	RI,MS
Vinyl hexanoate	986	RI,MS
6-Methyl-5-hepten-2-one	989	RI,MS
2,7,7-Trimethyl-3-oxatricyclo[4.1.1.0(2,4)]octane	991	RI,MS
β-Myrcene	991	RI,MS
2-Pentylfuran	992	RI,MS
cis,cis-4,6-Octadienol	994	RI,MS

Butyl butanoate	998	RI,MS
3-sec-Butylcyclohexene	999	RI,MS
Decane	1000	RI,MS
Octanal	1005	RI,MS
5-Methyldecane	1005	RI,MS
1,9-Decadiene	1009	RI,MS
β-Ocimene	1010	RI,MS
(E,E)-2,4-Heptadienal	1013	RI,MS
Hexanoic acid	1016	RI,MS
5-Methyl-5-hexenoic acid	1017	RI,MS
1-Isopropyl-4-methyl-1,3-cyclohexadiene	1018	RI,MS
Pent-2-en-4-ynyl cyclopropanecarboxylate	1020	RI,MS
Methyl (E)-4-heptenoate	1024	RI,MS
6-Methylenecycloocta-1,3-diene	1024	RI,MS
o-Cymene	1027	RI,MS
Methyl heptanoate	1030	RI,MS
1-Methyl-5-isopropenylcyclohexene	1031	RI,MS
1-Isopropenyl-4-methylenecyclohexane	1033	RI,MS
Eucalyptol	1034	RI,MS
2-Ethyl-1-hexanol	1035	RI,MS
2,2,6-Trimethylcyclohexanone	1038	RI,MS
3,5-Dimethyl-2-cyclohexen-1-one	1039	RI,MS
Benzyl alcohol	1042	RI,MS
1,5-Heptadiene-3,4-diol	1043	RI,MS
5-(3-Buten-1-yl)-1,3-cyclohexadiene	1043	RI,MS
3,5-Octadien-2-ol	1044	RI,MS

5-Ethenyldihydro-5-methyl-2(3H)-furanone	1046	RI,MS
Phenylacetaldehyde	1049	RI,MS
(Z)-3,7-Dimethyl-1,3,6-octatriene	1053	RI,MS
2,2,4,4-Tetramethyloctane	1057	RI,MS
γ-Terpinene	1062	RI,MS
3,3,6-Trimethyl-1,5-heptadien-4-ol	1064	RI,MS
2,2,7,7-Tetramethyloctane	1067	RI,MS
Acetophenone	1072	RI,MS
5-(Methylenecyclopropyl)pentanal	1073	RI,MS
(Z)-2-Octen-1-ol	1073	RI,MS
cis-Linalool oxide	1076	RI,MS
(E,E)-3,5-Octadien-2-one	1076	RI,MS
cis-2-Ethyl-2-hexen-1-ol	1077	RI,MS
cis-5-Ethenyltetrahydro-α,α,5-trimethyl-2-furanmethanol	1081	RI,MS
(2-Methyl-1-propenyl)cyclohexane	1083	RI,MS
3,7-Dimethyl-1,7-octadien-3-ol	1089	RI,MS
trans-Linalool oxide (furanoid)	1090	RI,MS
1-Methyl-4-(2-methyloxiranyl)-7-oxabicyclo[4.1.0]heptane	1092	RI,MS
Methyl benzoate	1097	RI,MS
4-Isopropylcyclohexanol	1099	RI,MS
Linalool	109999	RI,MS
1-Isopropenyl-3-propenylcyclopentane	1104	RI,MS
Nonanal	1105	RI,MS
Octahydro-6-methyl-3-methylenebenzofuran	1107	RI,MS
4-Hydroxy-4-methylcyclohexanone	1109	RI,MS
1,5,5-Trimethylbicyclo[2.2.1]heptan-2-ol	1110	RI,MS

Fenchol	1114	RI,MS
2-Phenylethanol	1117	RI,MS
(Z)-6-Nonenal	1118	RI,MS
trans,trans-5-Caranol	1120	RI,MS
Methyl octanoate	1128	RI,MS
8-Methyl-1-undecene	1135	RI,MS
2,3,5,8-Tetramethyldecane	1141	RI,MS
2-Bornanone	1150	RI,MS
4,4-Dimethyl-2-propenylcyclopentanone	1152	RI,MS
Citronellal	1160	RI,MS
Isoborneol	1161	RI,MS
Borneol	1170	RI,MS
Menthol	1177	RI,MS
2,2,6-Trimethyl-6-vinyltetrahydro-2H-pyran-3-ol	1179	RI,MS
Bicyclopentyl-1,1'-diene	1181	RI,MS
3-Pentenylbenzene	1182	RI,MS
Methyl phenylacetate	1182	RI,MS
2,2-Dimethyl-3-(2-propenyl)cyclopropanecarboxylic acid	1182	RI,MS
Methylcyclohexylacetate	1182	RI,MS
Methyl 2,8-nonadienoate	1187	RI,MS
2,6-Dimethyl-3,7-octadiene-2,6-diol	1192	RI,MS
α,α,4-Trimethyl-3-cyclohexene-1-methanol	1194	RI,MS
2-Propyl-1-heptanol	1195	RI,MS
2-Methyl-5-isopropenylcyclohexanol	1198	RI,MS
Methyl salicylate	1198	RI,MS
Dodecane	1199	RI,MS

2,6,6-Trimethyl-1,3-cyclohexadiene-1-carboxaldehyde	1202	RI,MS
Isopulegol	1206	RI,MS
2-Nonyl acetate	1214	RI,MS
Cyclooctanecarboxaldehyde	1216	RI,MS
Methyl decanoate	1224	RI,MS
2,6,6-Trimethyl-1-cyclohexene-1-carboxaldehyde	1225	RI,MS
Methyl nonanoate	1226	RI,MS
(Z)-3,7-Dimethyl-3,6-octadien-1-ol	1233	RI,MS
Isobornyl formate	1233	RI,MS
Hexyl 2-methylbutanoate	1238	RI,MS
1-Isopropyl-4-methylbicyclo[3.1.0]hexan-3-yl acetate	1240	RI,MS
cis-4-Isopropylcyclohexanemethanol	1242	RI,MS
2,7-Dimethyl-2,6-octadien-1-ol	1258	RI,MS
(E)-3,7-Dimethyl-2,6-octadien-1-ol	1258	RI,MS
5-Acetoxy-dihydro-5-methyl-2(3H)-furanone	1263	RI,MS
5-Methyl-5-propylnonane	1264	RI,MS
Ethyl salicylate	1276	RI,MS
4,6-Dimethyldodecane	1281	RI,MS
3-Ethylundecane	1282	RI,MS
4,4,8-Trimethyl-5-nonenal	1290	RI,MS
Menthyl acetate	1296	RI,MS
Tridecane	1299	RI,MS
2,6,10,10-Tetramethyl-1-oxaspiro[4.5]dec-6-ene	1304	RI,MS
1-Tridecene	1307	RI,MS
(2,2,6-Trimethylbicyclo[4.1.0]hept-1-yl)methanol	1309	RI,MS
1-(1-Hydroxybicyclo[2.2.2]oct-5-en-2-yl)ethanone	1312	RI,MS

5,6,7,7a-Tetrahydro-3,6-dimethyl-2(4H)-benzofuranone	1313	RI,MS
(Z)-5-Tridecene	1326	RI,MS
2,6,11-Trimethyldodecane	1336	RI,MS
2,7,10-Trimethyldodecane	1338	RI,MS
trans-4-Ethenyl-4-methyl-3-isopropenyl-1-isopropylcyclohexene	1343	RI,MS
2,6,6,9-Tetramethyltricyclo[5.4.0.0(2,8)]undec-9-ene	1359	RI,MS
1,2,3,4-Tetrahydro-1,1,6-trimethylnaphthalene	1364	RI,MS
Eugenol	1365	RI,MS
5-Hexyldihydro-2(3H)-furanone	1368	RI,MS
3-Methyltridecane	1370	RI,MS
2-Butyl-2-octenal	1376	RI,MS
α-Cubebene	1381	RI,MS
(Z)-3-Hexenyl hexanoate	1382	RI,MS
3-Methylenetridecane	1385	RI,MS
1-Tetradecene	1392	RI,MS
β-Bourbonene	1393	RI,MS
β-Longipinene	1397	RI,MS
Octahydro-3,8,8-trimethyl-6-methylene-1H-3a,7-methanoazulene	1397	RI,MS
Tetradecane	1399	RI,MS
6,10-Dimethyl-2-undecanone	1406	RI,MS
1-(2,2,5a-Trimethylperhydro-1-benzoxiren-1-yl)-2-buten-1-one	1408	RI,MS
Longifolene	1417	RI,MS
11,11-Dimethylspiro[2.9]dodeca-3,7-diene	1423	RI,MS
2-Methylene-4,8,8-trimethyl-4-vinylbicyclo[5.2.0]nonane	1430	RI,MS
4-(2,6,6-Trimethyl-2-cyclohexen-1-yl)-3-buten-2-one	1435	RI,MS
10,10-Dimethyl-2,6-dimethylenebicyclo[7.2.0]undecane	1439	RI,MS

cis-α-Bergamotene	1443	RI,MS
6,10-Dimethyl-5,9-undecadien-2-one	1456	RI,MS
4,11,11-Trimethyl-8-methylenebicyclo[7.2.0]undec-4-ene	1460	RI,MS
2,6,10-Trimethyltridecane	1462	RI,MS
cis-α-Bisabolene	1465	RI,MS
3-Methyltetradecane	1467	RI,MS
2-Methyl-5-oxo-1-cyclopenten-1-yl butanoate	1469	RI,MS
1,2,2,4,4,5,6,7-Octamethylbicyclo[3.2.0]hept-6-en-3-one	1475	RI,MS
cis-1,2,3,5,6,8a-Hexahydro-4,7-dimethyl-1-isopropylnaphthalene	1486	RI,MS
1-(1,5-Dimethyl-4-hexenyl)-4-methylbenzene	1488	RI,MS
4-(2,6,6-Trimethyl-1-cyclohexen-1-yl)-3-buten-2-one	1493	RI,MS
α-Muurolene	1509	RI,MS
α-Farnesene	1510	RI,MS
Decahydro-1,1,7-trimethyl-4-methylene-1H-cycloprop[e]azulen-7-ol	1517	RI,MS
3,7,11-Trimethyl-1-dodecanol	1525	RI,MS
Methyl dodecanoate	1525	RI,MS
4-Isopropyl-3,7-dimethyloctahydro-1H-cyclopenta[1,3]cyclopropa[1,2]benzen-3-ol	1527	RI,MS
7-Methylpentadecane	1528	RI,MS
1-Isopropyl-4,7-dimethyl-1,2,3,5,6,8a-hexahydronaphthalene	1532	RI,MS
cis-Calamenene	1534	RI,MS
cis-Hexahydro-8a-methyl-1,8(2H,5H)-naphthalenedione	1534	RI,MS
5,8-Diethyldodecane	1536	RI,MS
3,5,5,9-Tetramethyl-4a,5,6,7,8,9-hexahydro-2H-benzo[7]annulene	1542	RI,MS
3-Ethenyl-3-methyl-2-isopropenyl-6-isopropylcyclohexanol	1545	RI,MS
5,6,7,7a-Tetrahydro-4,4,7a-trimethyl-2(4H)-benzofuranone	1545	RI,MS
1,2,3,4,4a,7-Hexahydro-1,6-dimethyl-4-isopropylnaphthalene	1548	RI,MS

4-Ethyltetradecane	1551	RI,MS
5-Methylpentadecane	1552	RI,MS
4-Isopropyl-6-methyl-1-methylene-1,2,3,4-tetrahydronaphthalene	1555	RI,MS
3,7,11-Trimethyl-1,6,10-dodecatrien-3-ol	1568	RI,MS
3-Methylpentadecane	1570	RI,MS
(3E,7E)-4,8,12-Trimethyl-1,3,7,11-tridecatetraene	1582	RI,MS
1-Hexadecene	1592	RI,MS
Hexadecane	1599	RI,MS
8-Methylenepentadecane	1601	RI,MS
2-(4a,8-Dimethyl-3,4,4a,5,6,7,8,8a-octahydronaphthalen-2-yl)propan-2-ol	1615	RI,MS
(E,E)-1,5-Dimethyl-8-isopropylidene-1,5-cyclodecadiene	1620	RI,MS
2-Methyl-4-(2,6,6-trimethylcyclohex-1-enyl)-2-buten-1-ol	1622	RI,MS
2-(4a,8-Dimethyl-2,3,4,4a,5,6-hexahydronaphthalen-2-yl)propan-1-ol	1643	RI,MS
2-Hydroxy-2,4,4-trimethyl-3-(3-methyl-1,3-butadienyl)cyclohexanone	1654	RI,MS
1,6-Dimethyl-4-isopropylnaphthalene	1690	RI,MS
(Z)-7-Methyl-1-tetradecenyl acetate	1706	RI,MS
Methyl tetradecanoate	1726	RI,MS
2,3-Dimethylheptadecane	1770	RI,MS
Methyl 14-methylpentadecanoate	1825	RI,MS
Neophytadiene	1839	RI,MS
Phytol	2118	RI,MS
9-Octadecyne	1863	RI,MS
(Z)-8-Pentadecen-1-yl acetate	1884	RI,MS
Methyl hexadecanoate	1925	RI,MS
Isophytol	1948	RI,MS
cis-7-Hexadecenoic acid	1951	RI,MS

Methyl 9,12-octadecadienoate	2096	RI,MS
Methyl (Z)-6-octadecenoate	2102	RI,MS
Methyl (Z,Z,Z)-9,12,15-octadecatrienoate	2104	RI,MS
Methyl stearate	2130	RI,MS
